# Medical Device Development Process, and Associated Risks and Legislative Aspects-Systematic Review

**DOI:** 10.3389/fpubh.2020.00308

**Published:** 2020-07-30

**Authors:** Petra Marešová, Blanka Klímová, Jan Honegr, Kamil Kuča, Wan Nur Hidayah Ibrahim, Ali Selamat

**Affiliations:** ^1^Faculty of Informatics and Management, University of Hradec Kralove, Hradec Kralove, Czechia; ^2^Biomedical Research Centrum, University Hospital Hradec Kralove, Hradec Kralove, Czechia; ^3^Faculty of Computing, Universiti Teknologi Malaysia & Media and Game Innovation Centre of Excellence (MaGICX), Universiti Teknologi Malaysia, Kuala Lumpur, Malaysia

**Keywords:** medical devices, development, stages, risks, legislations

## Abstract

**Objective:** Medical device development, from the product's conception to release to market, is very complex and relies significantly on the application of exact processes. This paper aims to provide an analysis and summary of current research in the field of medical device development methodologies, discuss its phases, and evaluate the associated legislative and risk aspects.

**Methods:** The literature search was conducted to detect peer-reviewed studies in Scopus, Web of Science, and Science Direct, on content published between 2007 and November 2019. Based on exclusion and inclusion criteria, 13 papers were included in the first session and 11 were included in the second session. Thus, a total of 24 papers were analyzed. Most of the publications originated in the United States (7 out of 24).

**Results:** The medical device development process comprises one to seven stages. Six studies also contain a model of the medical device development process for all stages or for just some of the stages. These studies specifically describe the concept stage during which all uncertainties, such as the clinical need definition, customer requirements/needs, finances, reimbursement strategy, team selection, and legal aspects, must be considered.

**Conclusion:** The crucial factor in healthcare safety is the stability of factors over a long production time. Good manufacturing practices cannot be tested on individual batches of products; they must be inherently built into the manufacturing process. The key issues that must be addressed in the future are the consistency in the classification of devices throughout the EU and globally, and the transparency of approval processes.

## Introduction

Each year, a considerable amount of medical technologies are developed (1), and billions of crowns are invested in their development to meet the increasing demand for medical technology innovation (MDI). Research shows that extensive implementation of healthcare services worldwide is heavily dependent on medical technologies. According to the healthcare use statistics provided by Organization for Economic Co-operation and Development (OECD) (2), numbers of medical technologies are constantly rising. As a result, more healthcare technology needs to be developed (3). Innovative processes in the pharmaceutical industry appear every 10–20 years, while medical technology becomes outdated within months. Thus, new medical device development processes, which meet the needs of contemporary drug treatments, are currently being investigated and developed.

Nevertheless, there are only a few technologies and resources that penetrate the market. Medical device development (MDD) is expensive and risky. High risk of technology failure in the market leads to the question: Would it be appropriate to create a process or guide to assess healthcare technology at the beginning of the development process so that the development process and future impacts can be addressed on time? (4).

Currently, around 88% of corporations that develop medical device technologies are not able to provide considerable returns for their investors (5). Companies mainly concentrate on regulatory approval targets, without careful scheduling that considers establishing a less costly and more sustainable process (6). Therefore, well-prepared and well-thought launch strategies that capture inefficiencies in a timely manner and lower total costs are crucial in ensuring a successful product development process and satisfying stakeholder requirements.

Product development, from conception to release to market, is a very complex process (7, 8). It significantly relies on the application of exact processes that enable developers to optimally stage development, testing, validation, verification, and market release (9).

Current MDD processes have to respond to several process challenges (10, 11); projects seldom advance as scheduled, and often modifications are introduced during the course of project development and implementation (12). These processes do not respect the current legislative changes that are taking place at the European Union (EU) level. Risk analysis is mostly separately addressed, with respect to specific phases of MDD.

The MDD process has been satisfactorily described in literature; however, there is a lack of comprehensive models that would support design teams with different experiences and backgrounds. In general, published studies in this area either address the MDD with a specific focus on regulations (9, 13, 14) or provide proposals for various approaches to MDD (9, 15, 16).

This paper provides an analysis and summary of current research in the field of MDD methodologies, discusses the phases of MDD, and evaluates associated legislative and risk aspects.

## Methods

### Research Strategy

The systematic review is based on PRISMA guidelines (17, 18). The databases searched (by authors P.M. and W.N.) included Scopus (2007–2019) and Web of Science (2007–2019). In addition, legislative documents on the Research Topic, as well as the websites of medical companies dealing with the phases of MDD were explored. Keywords included the following collocations: “*medical device* AND *process* AND *development*” in Web of Knowledge and Scopus. Few more studies were found searching with the more specific keyword groups “*medical device* AND *process* AND *development* AND *investment evaluation*” and “*medical device* AND *stage development*.” A Boolean operator procedure was used in the search. The database was searched from 1 October 2019 until 20 November 2019.

### Research Questions

To achieve the objective of this review, the main research questions (RQ) were derived as follows:

RQ1 What are the phases of the MDD process?
RQ2 What are the regulation needs related to MDD?
RQ3 What are the risk factors in MDD?
RQ4 With which phases of the MDD process are regulation needs and risk factors associated?

### Article Selection and Data Collection

**Figure 1:**
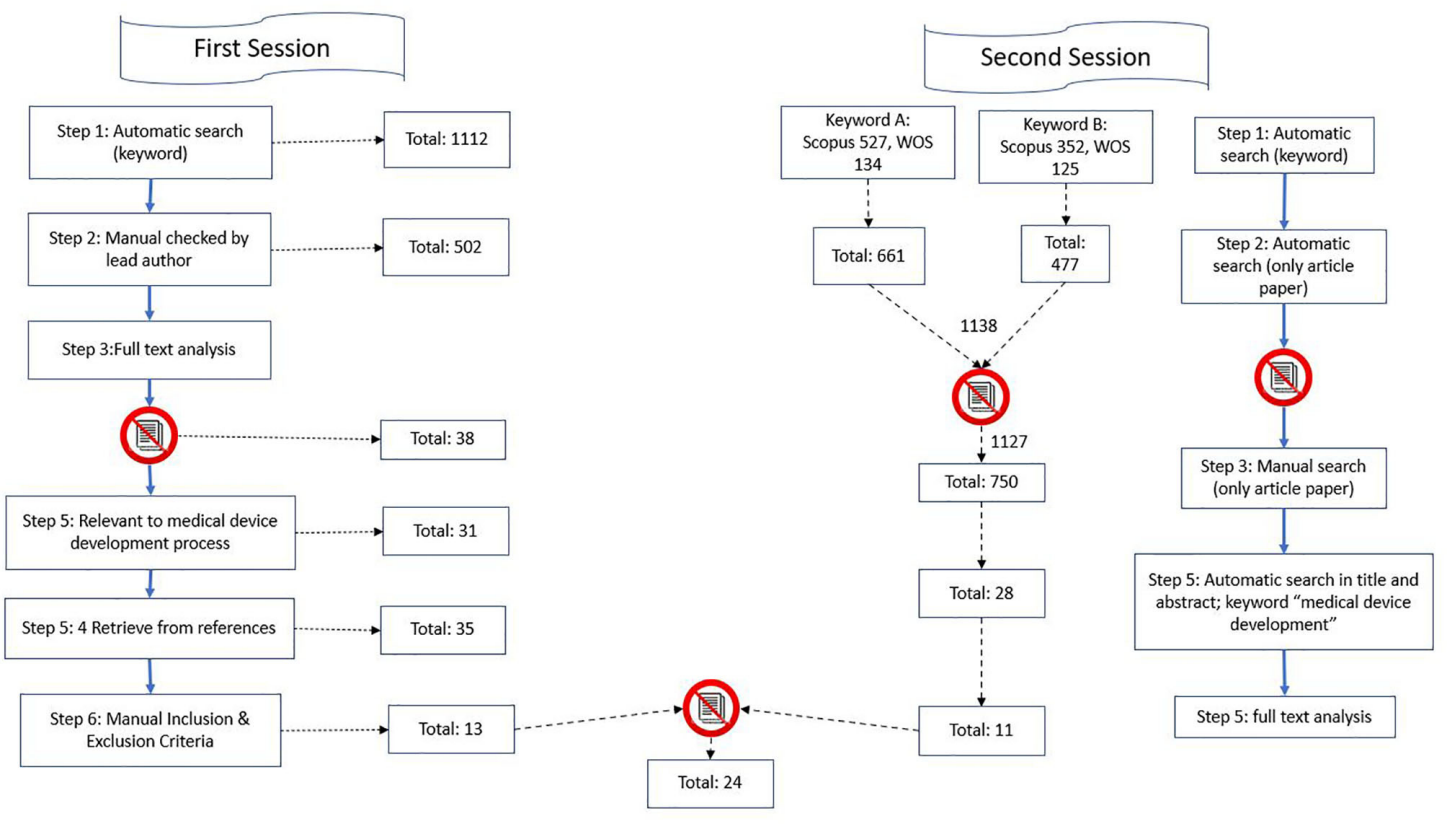
Illustration of search strategy.

The article selection process was divided into two sessions, and combined with an analysis, as shown in Figure 1. In the first session, we searched for publications between 2007 and 2017, and in the second session, we searched for articles published between 2017 and 2019.

#### The First Session

From the database/journal searches, 1,112 titles/abstracts were retrieved. The titles and abstracts of identified studies were checked by the lead author (J.K.) for relevance. Subsequently, the search was performed again, and it focused on the occurrence of at least one keyword in the title or abstract to significantly narrow down the selection. It provided the authors with a relevant entry-level file base. A total of 82 studies were found. The search procedure is illustrated below. As the search findings in:

Table 1 shows, most of the studies (19) were generated by the keyword string “medical device AND process AND development” in Web of Knowledge and Scopus. A few more studies were found by searching for a more specific keyword group: “medical device AND process AND development AND investment evaluation,” as well as by the search string “medical device AND stage development.”

**Table 1:** An overview of distribution of publications found in the first session.

**Keywords**	**WOS**	**ScienceDirect**	**Scopus**
Medical device process development	0	3	0
investment evaluation			
Medical device phase development	3	2	2
Medical device stage development	1	0	2
Medical device framework of development	6	0	8
Medical device process development	20	2	33

In cases of uncertainty, the full text of studies were checked for relevance. After removing duplicates and the titles/abstracts that were unrelated to the stages of the development process, we detected 38 peer-reviewed studies written in English. We included original articles and reviews. Of these, only 31 articles were relevant to the MDD process. These studies were investigated in full by BK and PM, with guidance from PM. Four more studies were detected from the references of the retrieved studies; thus, 35 articles were considered against our study's inclusion and exclusion criteria. On the basis of the criteria, 13 studies were included in the final analysis.

#### The Second Session

For articles published between 2017 and 2019, the search started with potential keywords based on the trends of the publication (Table 2). Two keywords were used as the main keywords that best corresponded to the objective of this research. Then, the details and abstract of the publication were extracted, and we agreed to narrow down the selection to articles from the database. Only articles that had the string “medical device development” in their title or abstracts were selected. A total of 28 papers were detected to fulfill the criteria. Thereafter, we performed manual full-text analyses, leaving 12 papers after the inclusion and exclusion criteria check. These 12 papers were combined with the 13 papers that we extracted from the first session. The whole process for the first and second sessions is illustrated in Figure 1.

**Table 2:** An overview of publication distributions for the second session.

	**Keyword A**	**Keyword B**
	“Medical device” AND process AND development	“Medical device development” AND innovation
Web of Science	144	125
Scopus	535	352
Total after duplication check	661	477

### Analysis

A combination of reviews and original studies were analyzed. Studies were selected on the basis of the following inclusion criteria:

I1 The publication date of the article is between 2007 and 2017.
I2 Reviewed full-text studies in scientific journals in English.
I3 The aim of the research is to suggest MDD processes.
I4 The study results proposed MDD processes or specifications associated with existing referenced phases of MDD.
I5 The study aimed to provide an overview of existing approaches in relation to risks and valid legislation.

The studies with the following attributes were gradually excluded from the analysis:

E1 The article was not written in English.
E2 The article did not mention the main string (“medical device development”) in its title or abstract.
E3 The article did not concern the research topic. For example,

- Cosgrove et al. (20) focused on a framework of key performance indicators to identify reductions in energy consumption in a medical device production facility;
- Songkajorn and Thawesaengskulthai (3) concentrated on one specific country and the development of medical devices according to the country's legislation;
- Cho and Kim (21) and Shaw (22) aimed at risk analysis;
- Songkajorn and Thawesaengskulthai (3) included incomplete data about the stages of MDD;
- Vaezi et al. (23) focused on the exploration of medical manufacturers' beliefs
- attitudes toward user involvement in the medical device design and development (24);
- Bruse et al. (25) focused on data analysis of image processing that will assist clinicians in decision making during MDD;
- Ciubuc et al. (26) focused on theoretical and experimental approaches to the detection of dopamine.
- The article described the development of healthcare software [e.g., (27, 28)].

E4 The distribution of publications based on their origin is shown in Table 3.

**Table 3:** The distribution of the publication based on origin.

**Country**	**UK**	**USA**	**Canada**	**Thailand**	**Portugal**	**India**	**Japan**	**German**	**Multiple origin**	**Total**
Total publication	4	7	1	1	1	1	2	1	6	24

### Text Analysis

**Figure 2:**
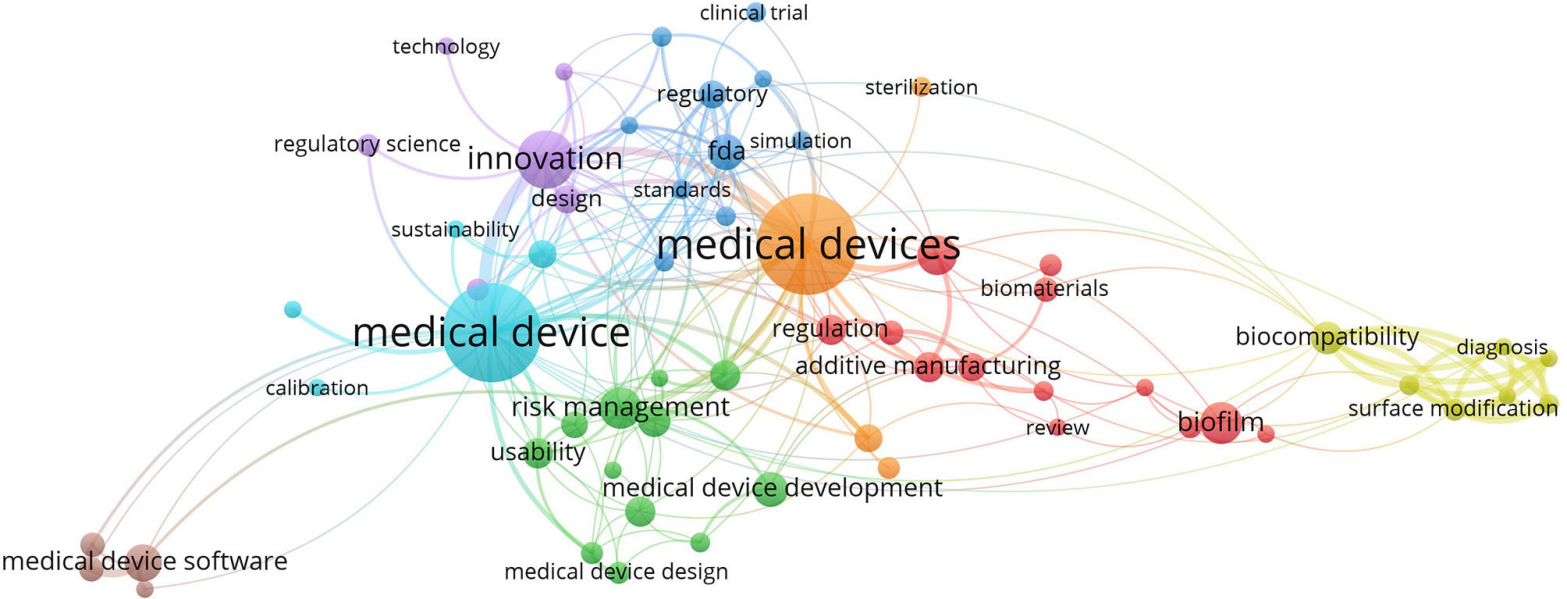
Keyword cluster for the second session; keyword A.

**Figure 3:**
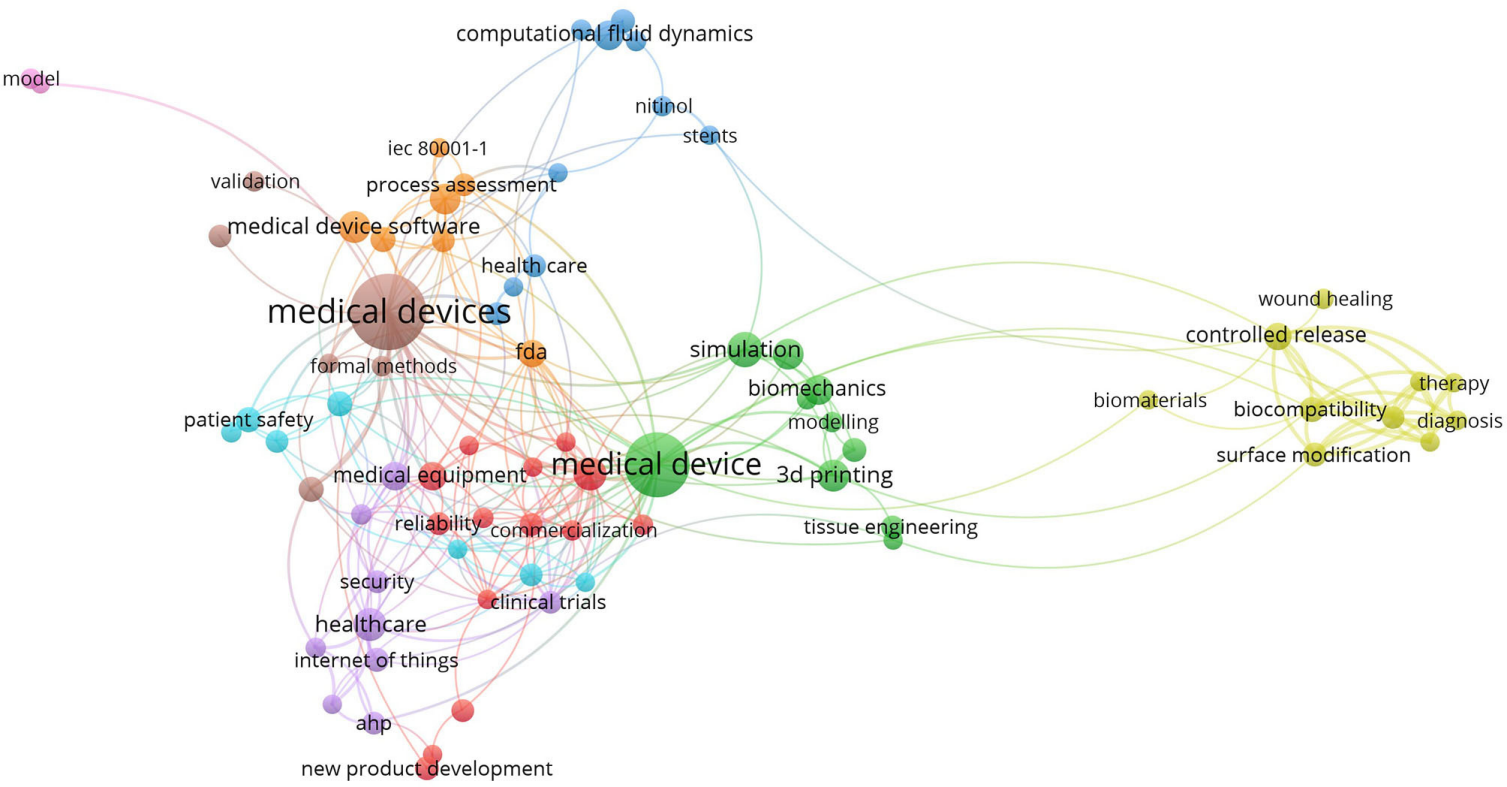
Keyword cluster for the second session; keyword B.

During the review process, text analyses were performed to assist the reviewer's decision. We used VOSviewer software to extract the relation between the co-occurrence of keywords before we decided on the keywords to be used in our search. Figures 2, 3 show the mapping of keyword co-occurrence for keywords A and B for the analysis of the second session (publications, 2017–2019).

#### Keyword Clusters

**Figure 4:**
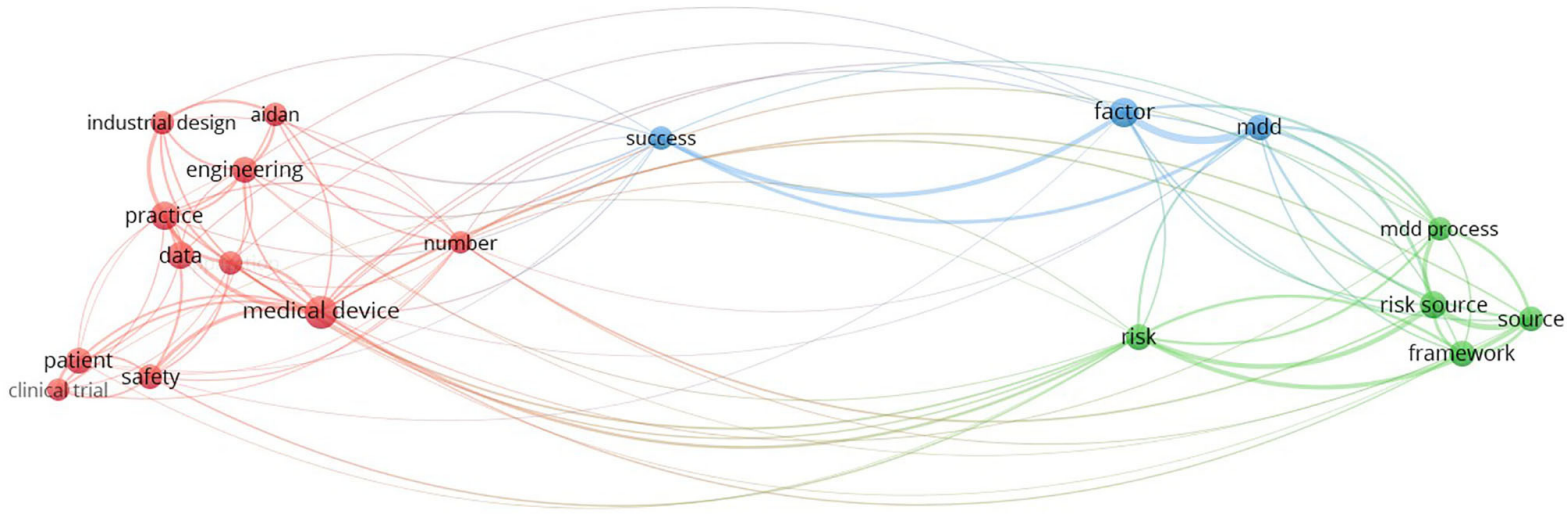
Title/abstract text-mapping in Step 5 (search strategy procedure).

Figure 4 shows the mapping of terms that co-occurred in the title and abstract during Step 5 of both sessions. A total of 61 non-duplicate publications were retrieved (35 from the first session and 28 from the second).

In the abovementioned figures, one can observe the areas that are solved in the publications. After excluding the topics and areas that are directly related to the technical solutions of MDD (e.g., represented by biomaterial, diagnosis, therapy, computational fluid dynamics), two main areas of study remained: regulatory/legislative aspects and risk/risk management. These two areas are further specified as they are related to the stages of MDD.

## Results

We detected a total of 13 research studies on the topic. Five of them originated in the United States, four in the United Kingdom, one in Canada, one in Thailand, and one in Portugal. One study was of multiple origin, i.e., USA, Canada, and Denmark. According to these studies, the MDD process comprises one to seven stages. Six studies (3, 9, 29–32) also contained a model of the MDD process for all stages or just some of the stages. The findings of the selected studies, especially the stages of the development of new medical devices, and presence or absence of relevant legislative aspects and risks, are summarized in Table 4 below. The columns are ordered according to the alphabetical name of the first author of the selected study. To minimize bias or systematic error, every time we combined the published paper, the duplication check is performed automatically using JaBref's software and manually based on author, title, and DOI. Other than that, each author also plays a role in checking either the classification of term or the content of each table, interpreting the real content published. Other than that, we are analyzing all the published articles and explain the process step-by-step in this article.

**Table 4:** Stages of MDD, an overview of the findings from the selected research studies.

**Study, year of publication, country of origin**	**Objective of the study**	**Stages/phases of the development of medical device**	**Legislative aspects (yes/no)**	**Risks (yes/no)**
USA, Canada, Denmark (33)	To discuss an agile method used for IT developments, especially in gating processes.	•Concept • Business case • Development • Testing • Launch	-	-
USA (19)	To describe best practices in early phase of medical development.	•Research and strategy phase • Product specifications	x	-
Canada (29)	To provide a new view on Heath Technology Assessment by sharing the risk associated with evaluations of effectiveness.	•Innovation and primary health research • R and D • Clinical assessment • Implementation • Assessment • System impact analysis and policy development, obsolescence/replacement	-	x
UK (30)	To develop a framework for valuing a novel medical device at the concept stage that balances benefit to the health care provider against commercial costs.	•Concept stage	-	-
Portugal (34)	To describe conceptual multifaceted framework for sustainable product development, as well as a MultiCriteria Hierarchical Model.	•Concept stage	x	-
UK (35)	To develop a technology confidence scale.	•Feasibility phase	x	x
UK (36)	To depict medical device development life cycle.	•Identification of user needs • Validation/ refinement of concept • Device design • Device evaluation		
USA (31)	To describe the complex nature of the medical device development process and its model.	•Clinical need definition and team formation • Feasibility, risk assessment, and conceptualization • Detailed design, verification, and validation • Production planning and qualification • Market introduction and post-launch	x	x
USA (37)	To illustrate the process of medical device development and its steps.	•Funding phase • Concept phase • Development phase • Verification and validation phase • Product phase • Market release phase	-	x
USA (9)	To describe a medical device development process from the initial concept stage to post-market surveillance.	•Initiation—opportunity and risk analysis • Formulation—concept and feasibility • Design and development—verification and validation • Final validation—product launch preparation • Product launch and post-launch assessment	x	x
USA (32)	To present a comprehensive stage-gate model to illustrate the investment process.	•General vision and investment strategy definition • Venture search • Screening and rapid pre-evaluation • Due diligence and negotiation of terms • Portfolio management • Evaluation and exit	x	-
Thailand (3)	To explore a medical device innovation development.	•Preliminary systematic analysis • Risk management • Conceptualized design • Clinical development • Production and MDI marketing	-	x
UK (24)	To suggest an acceptable and generic theoretical framework for involving various types of users in the medical device technology (MDT) development process (MDTDP).	•Concept stage • Device design • Testing and trial stage • Deployment stage	-	-
USA and EU (38)	To present the challenges in integrating human factor during design stage of MDD due implementing the standard	•Need analysis •Description of concept •Description of problem •Regulatory plan •Verification/ validation	x	x
German (39)	To introduce the adaption of Agile methods in developing product of medical technology.	•Preliminary phase (need analysis) • Iteration phase (tested regularly)—need of user and technical limitation • Final phase (risk and quality management evaluation.		x
Japan (40)	To outlines regulatory science in medical devices, taking into account differences from pharmaceuticals, and introduces specific initiatives.	•Research and development by physician - Trial manufacture of improved product - Variety of non-clinical study - Clinical use - Improvement of prototype • Development by company - Production of prototype - Variety of non-clinical study - Improvement of prototype - Feasibility study - Pivotal study • Application • Approval • Post-marketing surveillance	x	x
UK and USA (41)	To identify and explores risk sources in MDD process.	•Identification of MDD process risk source (literature and expert interview) • Pilot survey of identified risk source • Phase I ❖ Ranking of risk sources by experts ❖ List of MDD process risk sources • Phase II ❖ ISM methodology ❖ ISM-based model • Phase III ❖ Model validation ❖ Final model of MDD process risk sources.	x	x
USA and EU (42)	To synthesize a PDP model for SMEs in the specific medical sector, by incorporating the best practices of the engineering area and particularities of the medical area.	•Strategic Planning • Project Planning • Feasibility Study • System Design • Detailed Design • Production Process • Production Support • Product Launch • Monitoring • Discontinuance	x	x
USA (43)	To explore the consideration of patient and care partner perspectives during all aspects of development from design and clinical trials to regulatory approval.	•Device design • Clinical trials • Regulatory approved	x	x
Japan (44)	To proposed HFE/UE development process model incorporating human-centered design and safety design.	•Clarification and elaboration of the product development process, • Quantification of usability • Improvement of methods, tools, and environment		x
USA and EU (45)	To describe the framework of TED's continuing activities to advance research and clinical tools for TBI drug and device development.	•Prequalification stage	x	x
India (46)	To explore the success factors for MDD using literature review and opinions of experts.	•Evaluation phase		x
Mostly in India. But combine with USA, UK, Brazil, and China (47)	To model and prioritize risk sources in MDD process by using the combination of SEM and TOPSIS framework.	•Assignment of ratings to criteria and the alternatives • Compute aggregate fuzzy rating for criteria		x
USA (48)	To understand the design elements and the commercial requirements in developing new biomaterial in the market.	•Design Phase	x	x

The findings in Table 4 show that there is no agreement on the number of stages required for the development of medical devices. The number of product life cycle stages usually ranges from four to six. The study by Ocampo and Kaminski (42) suggests three stages—pre-development, development, and post-development (the PDP model). The specific medical device development is written in Table 4. Some studies, such as that of Girling et al. (30), Hede et al. (34), and Johnson and Moultrie (35), focus only on one stage of the MDD process, especially the concept stage during which all uncertainties such as the clinical need definition, customer requirements and needs, finances, reimbursement strategy, team selection, or legal aspects must be considered. The most representative studies are that of Medina et al. (31) and Pietzsch et al. (9), which include all key phases of the MDD life cycle, as well as legal aspects and risk factors; other studies are less detailed, and their model often lacks case studies [e.g., (32)]. Although Privitera et al. (38) reviews 18 medical devices as case studies and the reviews include legislative and risk analysis aspects, the research focuses only on the design stage of the MDD process. This is also true for the study by Panescu (37), whose descriptions of individual stages are very generic. He does not formulate a way to implement individual activities but only lists the activities and the order in which they should be performed. Moreover, the stages are not connected to specific legislative conditions or the type of medical device according to its level of risk.

According to Pietzsch et al. (9), the comprehensive MDD life cycle comprises five phases. Before the commencement of Phase 1, a clinical needs analysis must be conducted. Sometimes, this phase is referred to as Phase 0. Furthermore, preliminary market analysis must be conducted to check whether there is a satisfactory market opportunity for this clinical need and whether the new product is compatible with the company's strategy and ability to successfully commercialize this product. This phase is followed by Phase 1, with several important steps. These include a financial review and market analysis or competitive assessment that focuses on needs assessment and validation, demographics analysis, and SWOT analysis. These are followed by the legal intellectual property (IP) analysis and the regulatory review. The final step is to develop a business plan. In Phase 2, a cross-functional team is formed to formulate the concept, evaluate feasibility, and develop a design plan. Models and prototypes are made, and an initial design for manufacturing is developed. In addition, regulatory and reimbursement strategies from Phase 1 are further specified in this phase to comply with new requirements. In Phase 3, verification and validation tests are conducted to ensure that the quality of the device meets set standards and customer needs. In addition, regulatory and reimbursement activities continue in this phase. In Phase 4, formal design prints are made, and preparations are commenced for a medical device launch. The key step in this phase in the United States is the receipt of regulatory approval/clearance from the FDA. Phase 5 includes the product launch and post-market monitoring. If the device appears to succeed, it is distributed for widespread clinical use. Post-market activities involve post-market monitoring, quality audits, clinical validation, and the constant improvement of products and processes. Medina et al.'s (31) MDD stages resemble those of Pietzsch et al. (9). However, in comparison with Pietzsch et al.'s, they form the cross-functional team earlier on in Phase 1, while product launch preparation is a part of Phase 5.

Generally, the abovementioned linear stage-gate processes of the chosen authors have been used for almost three decades and been pivotal contributions for the medical device industry (49), because they are both conceptual and functional. Furthermore, they acknowledge that MDI is a manageable process (49).

Nevertheless, for the general model to be at least partially usable as a best practice, it must be updated to link to valid legislation, related risks, and valid changes in the management system of individual activities related to the audit trends and development of modern technologies, which affect most business activities.

## Discussion

The findings of the selected studies on the Research Topic show that the comprehensive MDD life cycle comprises five phases: opportunity and risk analysis phase, concept and feasibility phase, verification and validation phase, product launch preparation phase, and product launch and post-launch assessment phase. These individual MDD phases are linear and separated by gates that are characterized by certain set criteria that must be met before MDD can proceed further. That is why the whole MDD process is also called a linear stage-gate process, which is the most commonly used process in the development and innovation of medical devices.

However, Goldenberg and Gravagna (6) identified several gaps in the traditional stage-gate product development process. They point out that the stage-gate approach is linear, without a full life cycle plan and that companies, especially smaller ones, mainly focus on regulatory approval milestones than on providing significant returns to potential stakeholders. They suggest implementing an integrated customer engagement roadmap approach that identifies all stakeholder requirements/needs and device-specific marketing messages for product differentiation. Furthermore, detailed information on budget, timeline for data studies, and communications and marketing is included. Overall, a global launch strategy is implemented.

In addition, Cooper and Sommer (33) proposed the hybrid “agile-stage-gate” approach, which can be integrated into the traditional stage-gate model for the following benefits:

- It is built on customer needs in a cost-effective way.
- It reacts quickly to needs.
- It copes with uncertainty and ambiguity that are typical of innovative developments.
- It deals with resourcing issues more directly.

Furthermore, the sources of risks that can threaten the whole MDD process, in terms of price, timing, and quality, should be carefully considered to avoid failure. The key issue is meeting user needs. As far as the legislation aspects are concerned, the key issues are consistency in the classification of devices in the EU countries, as well as the transparency of the approval process worldwide.

### Risk Aspects

Individual MDD phases are closely connected with risks (50–54) that the individual steps bring about. For example, developing a new medical device is quite costly and risky (36); its success significantly relies on the application of accurate processes (9). Product designers and developers attempt to reduce these risks; however, tough competition encourages them to investigate the sources of risks during the MDD process, which can threaten the MDD process in terms of price, timing, and quality (38, 41). Aguwa et al. (55) reported that medical technology is quite unsuccessful (90%) during the first prototype test, which should be carefully considered by any MDD company. Some researchers have evaluated risks in medical device design. Privitera et al. (38) indicated the integration of human factors as one of the methods to reduce risks during the design stage of the MDD process; however, challenges exist because of the implementation of standards. These challenges can be solved if both parties, medical device developers and users, cooperate. Schmuland et al. (56) provided practical ideas to allow medical device manufacturers to evaluate residual risk of their devices. Risk analysis (ISO 14971) and failure analysis (FMEA) were combined by Chan et al. (57) to ensure device quality in the design phase of the MDD process, with a case study of a ventilation breathing circuit. Rane and Kirkire (41) summarized the key risks into three main groups: user-related sources of risks, internal sources of risks, and third party-related sources of risks. User-related risks include poor translation of user requirements or unmet user needs/requirements. Internal risks are due to the lack of application of adequate standards to check device performance; poor consideration of the effect of labeling and packaging; or poor communication among device developers, end users, and marketing. Third party-related sources of risks may include lack of training for end users; improper or poor assessment of progress by reviewers; and poor planning for regulatory and clinical approvals and tests. Their findings indicate that the most important source of risks is unmet user needs, which means that user needs should be met to successfully market any device.

The detection of risks and their sources in the MDD process plays a significant role, because it can prevent a lot of adverse effects of the use of medical devices by end users, save a lot of time on design and development of the medical device, and reduce costs during the MDD process. Therefore, the MDD process should be critically planned and modeled to decrease the number of risks and their severity.

### Legislative Aspects

Global harmonization in the field of medical device regulation is following the pathway set by the pharmaceutical industry at the turn of the 1980s (58). In 1989, regulatory bodies of the United States, EU, and Japan came to the conclusion that it would be more effective for the industry to develop universal standards for all aspects of drug development, manufacturing, and pharmacovigilance, with the aim to bring more safety to the process of drug manufacturing (Table 5).

**Table 5:** The regulation in the European Union and in the United States of America.

		**EU**		**USA**	
Risk Classification	Low risk	Class I—low risk		Class I	
	Medium risk	Class IIa Class IIb Class Is—sterile		Class II	
		Class Im—with measuring function)			
		Class IIb			
	High risk	Class III		Class III	
Risk Management				ISO 14971	
Quality System		EN ISO 13485:2016		cGMP (21 CFR part 820)	
-Exemption		Non-sterile low risk (declaration of conformity)	Class I	Some types—low risk nonsterile−510(k) with asterisk	
Inspection organizations		Accredited Notified Bodies		US FDA–CDRH	
Competent authority		Member states Competent Authority		US FDA–CDRH	
Clinical Investigation		Notify Competent Authority in state where is conducted		Non-substantial Risk devices—Institutional Review Board approval	Class I and some Class II
				Substantial Risk Devices—FDA Granted Investigational device exemption and IRB approval	Class II and Class III
Market Authorization	Low Risk	Self-Declaration of Conformity—CE	Class I	No FDA scrutiny needed	Class I and some Class II devices
	Medium Risk	Quality management system in place, Technical Construction File and Device Dossier Review by NB	Class Is, Im, IIa,	510(k)—Substantially equivalent to legally marketed product	Vast majority of Class II and some Class III
	High Risk	ditto	Class IIb, III	Pre-Market Authorization—Thorough check on safety by TCF, preclinical and clinical data review	Vast majority of Class III
Post marketing Vigilance	Report of serious public health threat	Within 2 days		Within 5 days	
	Report deaths or serious injuries	Within 10 days		Within 10 days	
	Other	Within 30 days		Within 30 days	

Therefore, the International Council^1^ for Harmonization of Technical Requirements for Pharmaceuticals for Human Use (ICH) was founded. Since then, almost all important pharmaceutical markets have closely similar legislation that stems from ICH guidelines. ICH plays a crucial role in adopting novel policies for the safety of pharmaceuticals.

The Global Harmonization Task Force (GHTF), founded in 1992, was replaced in 2011 by the International Medical Device Regulators Forum (IMDRF). In an ever-changing global market, focus on harmonization is needed to achieve the desired level of safety of medical devices. During the 1980s, almost no regulation of medical devices existed. Since the 1990s, some regulations have emerged mainly in the United States and the EU, as well as in the East Asia region, mainly in Japan and Taiwan. Since the beginning of the new millennium, one can observe convergence in the regulation of the medical devices industry owing to the work of the GHTF. However, a global world needs global approaches. That is why the IMDRF came to life. The two largest markets for medical devices are Europe and North America. Regulatory requirements converge on both sides of the Atlantic; yet, American rules had been stricter compared to European rules—until the recent approval of the new medical devices regulation (MDR) by the European Parliament. The rules concerning medical devices had been much more relaxed in the EU; however, after the large-scale scandal involving Poly Implant Prothèse (PIP)-manufactured breast implants, the European Union embarked on the path leading to the approval of the MDR. As thorough as it is, it is still inferior to Title 21, Part 812 and 820 of the Code of Federal Regulations set by the United States, also referred to as current good manufacturing practices (cGMP).

In the EU, the key role is versed on the so-called “accredited notified bodies” that are privately held for-profit companies. Their nature poses a great risk for the whole system. Since there is only a limited number of such bodies (gradually decreasing), and because of the mandatory re-evaluation of all medical devices approved in the EU common market, there will be shortages of available capacity for re-evaluation. Simultaneously, notified bodies would probably be less willing to inspect small companies, which make only a few types of medical devices and tend to be generally less prepared for the transition to novel regulations, because it will be much profitable to inspect large companies with diverse portfolios and better prepared paperwork.

Another limiting factor is the relatively large number of such bodies compared to the situation in the United States where all inspectors are employees of the Food and Drug Administration (FDA), and partially the Center for Devices and Radiological Health (CDRH). It seems plausible that there could be significant differences between the level of scrutiny among bodies based in distant parts of the EU. As such, the key factor of proposed regulation could be endangered by this flaw. Another issue that is addressed by the MDR is post-marketing vigilance of medical devices. The novel regulations impose the duty of post-marketing follow-up for all devices marketed in the EU.

Since the beginning of the PIP breast implant scandals, there has been a steady shift in the perception of how to achieve this goal within the industry. Before the MDR came into effect, the focus had been more on the safety of individual products. Thus, almost all effort was put to releasing the product by obtaining the CE mark. However, as a lesson learned from the pharmaceutical industry, safety should be achieved primarily by setting up a rigorous framework of rules for the whole product life cycle. A quick overview of the regulations in the EU and the United States could be seen in Table 5. The EU and the United States were chosen because other states are modeling regulations after theirs. For further information on the topic, readers are kindly referred to the reviews by Gupta and Thomke (10) and Ocampo and Kaminski (42), which discuss the global regulation aspects of medical devices.

In Japan, as stated in Niimi (40), the risks are divided into four classes: Class I, Class II, Class III, and Class IV, where the highest risk is in Class III and Class IV, which are for highly controlled medical devices and need the approval from the minister and a review by the Pharmaceutical and Medical Devices Agency (PMDA).

## Conclusion

Regarding the phases involved in MDD, and the related regulations and risk factors, the results indicate that the general model applied in the MDD process should follow the well-established linear stage-gate process, which is conceptual and manageable from the perspective of innovation. Nevertheless, the model should include recently suggested approaches such as implementing an integrated customer engagement roadmap. In addition, the model must respond to current valid legislation processes, their changes, and related risks, as well as to the valid changes in the management system of individual activities related to the audit trends and development of modern technologies, which affect most business activities. The crucial factor in healthcare safety (59, 60) is the stability of factors over a long production time. Good manufacturing practices cannot be tested on individual batches of products; they must be inherently built into the manufacturing process. This is the goal that medical device regulations and cGMP are trying to achieve. The key issues that must be addressed in the future are consistency in the classification of devices throughout the EU and globally, and the transparency of the approval processes.

## Strengths and Limitations of this Study

- This review presents in-depth specifications of the stages of the medical device development process and the associated risks, which are not described in organizational or managerial research. It provides a general point of view as opposed to large numbers of case studies.
- Research findings are strategically important for healthcare development, because they clearly state the requirements for medical device development and offer a way for researchers to apply this specific process in general managerial research.
- This study is limited in the sense that it cannot cover all consequences of changes in legislative aspects.

## Author Contributions

PM and KK suggested the design of the study. WI wrote the methodology. WI and PM searched the databases. PM, AS, BK, JH, and KK prepared the tables, wrote the manuscript, and reviewed the paper. All authors approved this version of the paper.

## Conflict of Interest

The authors declare that the research was conducted in the absence of any commercial or financial relationships that could be construed as a potential conflict of interest. The handling editor declared a past co-authorship with the author KK.

## Footnotes

^1^Between 1990 and 2015, it was known as the International Conference on Harmonization.

## Abbreviations

MD: Medical device
MDD: Medical device development
FDA: Food and Drug Administration
CDRH: Center for Devices and Radiological Health
GHTF: The Global Harmonization Task Force
MDR: International Medical Device Regulators Forum
MDR: Medical Devices Regulation
cGMP: Current Good Manufacturing Practices
FMEA: Failure analysis
TOPSIS: Technique for Order Performance by Similarity to Ideal Solution
SEM: Structural Equation Modeling.
